# *Toxoplasma gondii* and *Neospora caninum* infections in sheep and goats in Switzerland: Seroprevalence and occurrence in aborted foetuses

**DOI:** 10.1016/j.fawpar.2022.e00176

**Published:** 2022-08-17

**Authors:** Walter Basso, Fabienne Holenweger, Gereon Schares, Norbert Müller, Lucía M. Campero, Flurin Ardüser, Gaia Moore-Jones, Caroline F. Frey, Patrik Zanolari

**Affiliations:** a Institute of Parasitology, Vetsuisse-Faculty, University of Bern, Länggassstrasse 122, 3012 Bern, Switzerland; b Clinic for Ruminants, Vetsuisse-Faculty, University of Bern, Bremgartenstrasse 109a, 3012 Bern, Switzerland; c Friedrich-Loeffler-Institut, Federal Research Institute for Animal Health, Institute of Epidemiology, Südufer 10, 17493 Greifswald-Insel Riems, Germany; d Immunoparasitology Laboratory, Faculty of Veterinary Sciences, National University of La Plata, 60 y 118, 1900 La Plata, Argentina; e National Scientific and Technical Research Council (CONICET), Argentina; f Institute for Fish and Wildlife Health (FIWI), Vetsuisse-Faculty, University of Bern, Längassstrasse 122, 3012, Bern, Switzerland

**Keywords:** *Toxoplasma gondii*, *Neospora caninum*, Abortion, Serology, Zoonosis, PCR

## Abstract

*Toxoplasma gondii* and *Neospora caninum* infections are important causes of abortion in ruminants. Besides, meat from *T. gondii* infected animals represent a major infection source for humans. The occurrence of these protozoan parasites in Switzerland was investigated both, in a nationwide cross-sectional serological survey, and by molecular methods in aborted sheep and goat foetuses. A total of 653 sheep from 143 farms and 748 goats from 164 farms were tested by commercial ELISAs and inconclusive results were defined by immunoblot. Besides, a risk factor analysis for seropositivity was performed. The observed seroprevalences for *T. gondii* in sheep and goats were 66.3% and 50.5% at the animal level, and 90.9% and 81.1% at the farm level, respectively. For *N. caninum*, the detected seroprevalences in sheep and goats were 0.8% and 0.9% at the animal level, and 2.8% and 1.8% at the farm level, respectively. Older small ruminants, and sheep (vs. goats) had a higher risk of being seropositive to *T. gondii.* Alpine grazing in summer was identified as a protective factor for seropositivity to *T. gondii* in both animal species. *Toxoplasma gondii* and *N. caninum* DNA were detected in 6.1% and 2.4% (*n* = 82), and in 6.8% and 1.4% (*n* = 73) of the tested ovine and caprine foetuses, respectively. These results suggest the involvement of these parasites in abortions and reveal a high prevalence of *T. gondii* and lower prevalence of *N. caninum* infections in small ruminants in Switzerland. They also suggest that consumption of undercooked meat from *T. gondii* infected sheep and goats may represent a risk for public health.

## Highlights

• *Toxoplasma gondii* infections are very frequent in Swiss small ruminants.
• Meat from infected small ruminants represents a source for *T. gondii* human infection.
• Sheep have a higher risk of *T. gondii* seropositivity than goats.
• *Neospora caninum* seroprevalences in Swiss sheep and goats are very low.
• *T. gondii* and *N. caninum* were detected in ovine and caprine abortions in Switzerland.
• *T. gondii* and *N. caninum* should be considered in diagnosis of small ruminant abortion.

## Graphical abstract

**Figure:**
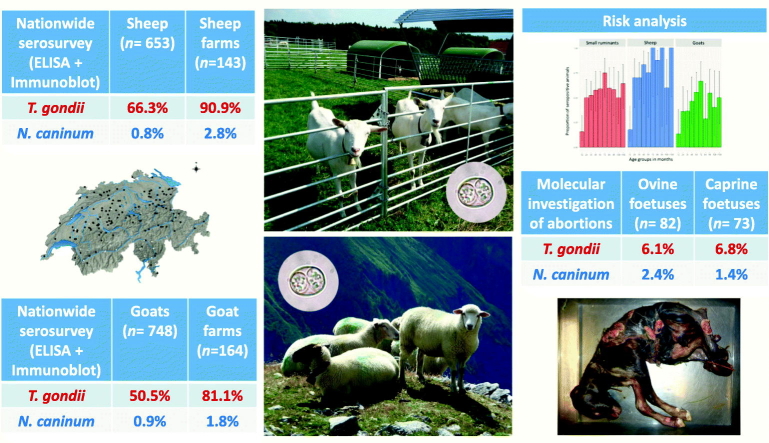

## 1 Introduction

*Toxoplasma gondii* and *Neospora caninum* (Apicomplexa, Sarcocystidae) are obligate intracellular protozoan parasites that can affect different warm-blooded species around the world. Both parasites have heteroxenous life cycles with carnivores as definitive hosts and represent important causes of reproductive disorders and economic loss in livestock production. Furthermore, meat from *T. gondii* infected animals is regarded as an important infection source for humans (Dubey et al., 2017; Dubey, 2022). Both parasites undergo an intestinal cycle in their definitive hosts (i.e., *T. gondii* in domestic and wild felids, *N. caninum* in dogs, wolves, coyotes, and dingoes), which are responsible for environmental contamination by shedding oocysts with the faeces during few weeks after infection. Small ruminants may become infected with both parasite species either horizontally, through ingestion of sporulated oocysts contaminating grass, fodder, or water, or vertically, by transplacental transmission from the dam to the foetus (Dubey, 2022; Dubey et al., 2017). After an acute phase of parasite multiplication as tachyzoites in potentially all tissues (including placenta and developing foetuses in pregnant animals), the infection leads to seroconversion and turns into chronicity, with development of tissue cysts containing bradyzoites, mainly in muscle and nervous tissues (Dubey, 2022; Dubey et al., 2017).

*Toxoplasma gondii* infection in non-pregnant ewes and goats is generally asymptomatic, or may present with unspecific clinical signs such as fever, nasal discharge, cough, tachypnoea and loss of appetite for few days, which may remain unnoticed in conventional production farms (Glor et al., 2013). However, primoinfection during gestation may lead to foetal death, abortion, mummification, stillbirth, or birth of weak lambs, depending on the time point of infection. Chronically infected ewes generally do not transmit *T. gondii* to future generations, as reactivation of an endogenous infection leading to transplacental transmission is thought to be infrequent in sheep (Buxton et al., 2007; Dubey, 2009). However, repeated abortions associated with *T. gondii* infection have been observed in goats (Dubey, 1982). *Neospora caninum* is considered one of the most important causes of infectious abortion in cattle worldwide, but also small ruminants, South American camelids and cervids may be affected (Dubey et al., 2017; Lindsay and Dubey, 2020). Reactivation of chronic *N. caninum* infection during pregnancy and endogenous transplacental transmission of the parasite to the offspring is known to be a common event in cattle, but the information on the efficiency of vertical transmission in small ruminants is still limited (Lindsay and Dubey, 2020). Nevertheless, there is initial evidence that this way of transmission is also efficient in the ovine and caprine hosts (de Oliveira Junior et al., 2020; Feitosa et al., 2021; González-Warleta et al., 2018; Pereira et al., 2021; Sánchez-Sánchez et al., 2021b).

Several studies on *T. gondii* and *N. caninum* infections in small ruminants have been carried out in Europe, but information about the occurrence of these parasitoses in Switzerland was limited (Wyss et al., 2000; Chanton-Greutmann et al., 2002; Hässig et al., 2003; Berger-Schoch et al., 2011; Frey et al., 2012).

The aims of this study were (i) to estimate the occurrence and distribution of *T. gondii* and *N. caninum* infections in sheep and goats in Switzerland in a first nationwide cross-sectional serosurvey, (ii) to identify risk factors, which may favour infection, and (iii) to assess the occurrence of these protozoan parasites in sheep and goat abortions.

## 2 Material and methods

### 2.1 Serological survey

#### 2.1.1 Animal sampling and farm data

Blood samples from sheep and goats were collected between May 2017 and September 2018 from 25 of the 26 Swiss cantons (with one canton, Basel-Stadt, having no registered farms). For both small ruminant species, farms were randomly selected and stratified according to the sheep or goat population size of the 25 sampled cantons. The average size of the sampled sheep and goat flocks were 47.4 (range 2–500) and 19.4 (range 2–173) animals, with median values of 29 and 10 animals, respectively. From each participating farm, 2 to 8 animals (mean = 4.6) were randomly selected, and basic information about the farm and sampled animals (i.e., small ruminant species, breed, and age) was immediately obtained after successful blood withdrawal from the *vena jugularis*. Subsequently, serum was extracted and stored at −20 °C until analysis.

A total of 1401 serum samples from 307 farms were obtained (i.e., 653 sheep samples from 143 farms and 748 goat samples from 164 farms; five farms bred both sheep and goats). This represents 0.2% and 1% of the total sheep (*n* = 342,163 animals on 8315 farms) and goat (*n* = 74,877 animals on 6364 farms) populations in Switzerland, as well as 1.7% and 2.6% of sheep and goat farms at the beginning of sampling, respectively (Erdin, 2017) (https://www.sbv-usp.ch/de/agristat-aktuell-09-17-der-nutztierbestand-der-schweiz/).

For statistical analysis, the animals were stratified into nine different age groups at yearly intervals with a tenth group including all animals older than 10 years. Animals aged 12 months and younger were considered “young animals” whereas older animals were considered “adult animals”. For 63 sheep and 114 goats, no date of birth was obtained. These animals were not considered in the risk factor analysis.

In order to assess possible risk factors for seropositivity, and thereof possible infection with *T. gondii* or *N. caninum,* a standardised web-based questionnaire was prepared in three of the four Swiss national languages (i.e., German, French and Italian) and sent to the participating farms via e-mail. The questionnaire contained further questions about the farm, livestock, management, presence of cats/dogs in the stables and pasture, problem with rodents, presence of other animal species in the farm, water supply, feed and feed storage, and about the occurrence of abortions in the herd (Suppl. Table 1). Responding to the questionnaire was voluntary and no submitted information was distributed to third parties. All collected data, as well as the subsequent evaluations were processed confidentially, and farmers were not informed about the serological status of their farms in advance.

The study was carried out in accordance with the Swiss animal welfare legislation (approval number BE 5/17+) and authorised by the involved cantonal animal welfare committees. Written informed consent was obtained from all farmers for enrolling sheep and goats in the study and for the publication of the results thereof.

#### 2.1.2 Serological tests

##### 2.1.2.1 ELISA

All serum samples were processed by indirect ELISAs to detect antibodies against *T. gondii* and *N. caninum* using two commercial kits: ID Screen® Toxoplasmosis Indirect Multi-Species (TOXO-MS; ID.vet, Grabels, France), validated for antibody detection in dogs, cats, goats, sheep, cattle and pigs, and ID Screen® *Neospora caninum* Indirect (NCS; ID.vet, Grabels, France), validated for detection of antibodies against *N. caninum* in serum, plasma or milk from cattle, sheep or goats. The TOXO-MS ELISA uses the P30 *T. gondii* tachyzoite surface protein as antigen, a multi-species-horse radish peroxidase (HRP) conjugate as secondary antibody and 3,3′,5,5′-tetramethylbenzidine (TMB) as substrate. In the NCS ELISA the antigen is based on a purified *N. caninum* extract. It uses an anti-ruminant conjugate as secondary antibody, and TMB as substrate. The tests were performed as indicated by the manufacturer. In both assays, a sample-to-positive ratio (S/P%) was calculated for each serum sample according to $S/P\%=\frac{{\mathrm{OD}}_{\mathrm{sample}}-{\mathrm{OD}}_{\mathrm{NC}}}{{\mathrm{OD}}_{\mathrm{PC}}-{\mathrm{OD}}_{\mathrm{NC}}}\;x\;100$, where OD is the optical density either of the sample, the positive controls (PC) or the negative controls (NC) of the kits. According to the manufacturer, animals with S/P% ≤ 40% were considered negative, inconclusive if 40% < S/P% > 50% and positive if S/P% ≥ 50%.

##### 2.1.2.2 Immunoblot

All sheep and goat samples with inconclusive results in *T. gondii* or *N. caninum* ELISA were subsequently tested by *in-house* immunoblots in order to define their serological status.

Immunoblot analyses based on the affinity-purified, native *T. gondii* tachyzoite surface antigen TgSAG1 (P30) and on the *N. caninum* tachyzoite antigens were essentially performed as recently described (Basso et al., 2020) with the exception that different secondary antibodies were used – i.e., donkey-anti-sheep IgG (whole molecule) peroxidase conjugate A 3415 (Sigma-Aldrich, St. Louis, USA) and rabbit-anti-goat IgG (whole molecule) peroxidase conjugate A 4174 (Sigma-Aldrich, St. Louis, USA), both at 1:600 dilution in PBS-Tween – for antibody detection in sheep or goat samples, respectively.

For *T. gondii*, a serum reaction was recorded as positive, if a single band of a relative molecular mass of 30 kDa was visible. The TgSAG1 antigen proved to be adequate for diagnostic of experimental and natural *T. gondii* infections in small ruminants and other animal species either by immunoblot or ELISA techniques in previous publications (e.g., Basso et al., 2020; Wyss et al., 2000; Sager et al., 2003; Tzanidakis et al., 2012).

For *N. caninum*, a serum reaction was considered positive when reaction against at least two out of five *N. caninum* relevant immunodominant antigens (i.e. 17, 29, 30, 33 and 37 kDa) was observed (Basso et al., 2020; Staubli et al., 2006; Wolf et al., 2005). If only one band was visible, the sample was considered inconclusive.

Serum samples from sheep experimentally infected with *T. gondii* (*n* = 6) as well as from naïve sheep (*n* = 3) (Glor et al., 2013) were included in the ELISA (in addition to the positive and negative control sera provided with the kits), and immunoblot analyses. Besides, control sera from sheep naturally infected with *N. caninum* (*n* = 1) and naïve to *N. caninum* (*n* = 1), as well as from goats naturally infected with *T. gondii* (*n* = 1) and *N. caninum* (*n* = 1) and naïve to both parasites (*n* = 2) from the Diagnostic Laboratory of the Institute of Parasitology, University of Bern (IPB) were also included.

### 2.2 Risk factor analysis

An analysis to identify risk factors associated with *T. gondii* and *N. caninum* seropositivity was performed with the collected information about the farms and the animals. A bivariable-multilevel-modelling (generalized linear mixed modelling fit by maximum likelihood [Laplace approximation]) approach was applied using the R statistical software (https://www.r-project.org/; Version 3.3.1) and in particular, the package lme4 (Bates et al., 2015). The animal serostatus (i.e., “seropositive” or “seronegative”) for *T. gondii* or *N. caninum* was considered as the dependent variable and the potential risk factors as independent variables. As individual animals clustered in farms, “farm” was included as a random effect variable in modelling seropositivity. Due to the finding that seropositivity clearly increased with age, data on “age” of the individual animals was included into each of the calculated models as an important effect-modifying explanatory variable, and these models were always calculated as bivariable, i.e., “age” (young [12 months and younger], adult [> 12 month]) was always considered together with the risk factor to be analysed. Animals with no information on birth date (sheep *n* = 63, goats *n* = 114) were excluded from the analysis. The Akaike information criterion (AIC) was used to characterize the relative model quality. This modelling strategy was previously used by Lücht et al. (2019) and Basso et al. (2020) and adapted for this study.

### 2.3 Real-time PCR investigation of aborted ovine and caprine foetuses

A total of 155 aborted foetuses (i.e., 82 ovine and 73 caprine foetuses) submitted to the IPB between 2010 and 2021 were tested by real-time PCR for *T. gondii* (Reischl et al., 2003) and *N. caninum* (Müller et al., 2002), which amplify the 529-bp repeat element of *T. gondii*, or the repetitive genomic sequence Nc5 of *N. caninum*, respectively. For this, DNA was extracted from brain samples using a commercial kit (Dneasy Blood & Tissue Kit, QIAGEN) as indicated by the manufacturer and processed by real-time PCR as previously described.

## 3 Results

### 3.1 Serological survey

#### 3.1.1 Detection of antibodies against *Toxoplasma gondii*

According to the different thresholds suggested by the manufacturer, 428/653 sheep were tested positive, 214/653 negative and 11/653 were inconclusive for *T. gondii* in ELISA. Besides, 375/748 goats yielded positive results, 348/748 negative and 25/748 were inconclusive. The seroprevalence at the animal level was found at 65.5% (CI 95%: 61.8–69.2%) for sheep and 50.1% (CI 95%: 46.5–53.8%) for goats, not including animals, which tested inconclusive. At the farm level, the ELISA seroprevalence of *T. gondii* was 90.9% (CI 95%: 85.0–95.1%) (130/143) for sheep farms and 81.1% (CI 95%: 74.3–86.8%) (133/164) for goat farms, respectively. All serum samples with inconclusive ELISA results were subsequently tested by immunoblot to define the serostatus. A total of 5/11 sheep samples and 3/25 goat samples with inconclusive ELISA results yielded positive results in immunoblot. These eight additional seropositive animals originated from farms that were already seropositive at the farm level.

Considering immunoblot results, the adjusted seroprevalence of *T. gondii* antibodies at the animal level in sheep was of 66.3% (433/653; CI 95%: 62.5–69.9%) and in goats of 50.5% (378/748; CI 95%: 46.9–54.2%). The seroprevalence at the farm level remained unchanged. The obtained values indicate apparent prevalences according to the sampled population and diagnostic tests used. A calculation of the true prevalence rates, referred to the whole small ruminant population was not attempted.

**Fig. 1:**
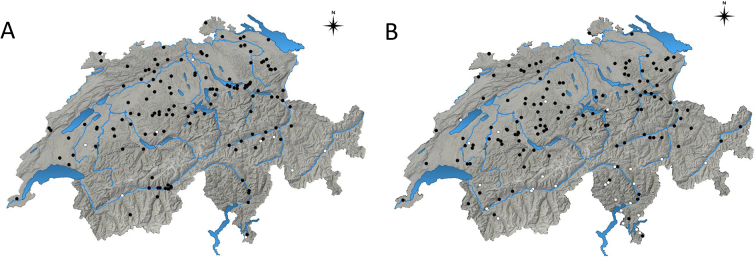
Geographical distribution of all tested farms breeding sheep (A) and goats (B) in Switzerland, indicating the farms in which seropositive animals for *Toxoplasma gondii* were detected (black dots) or not detected (white dots).

The geographical distribution of all the tested sheep (A) and goat (B) farms in Switzerland, indicating the farms in which seropositive animals for *T. gondii* were detected is displayed in Fig. 1

#### 3.1.2 Detection of antibodies against *Neospora caninum*

According to the cut-off suggested by the kit's manufacturer, 5/653 sheep were tested positive, 647/653 negative and 1/653 inconclusive for *N. caninum* antibodies. Besides, 7/748 goats showed positive, 741/748 negative and 0/748 inconclusive ELISA results.

The immunoblot analysis of the inconclusive sheep serum sample showed a negative result, and since no goat sample yielded inconclusive results in ELISA, the seroprevalences at the animal and farm levels estimated by ELISA remained unchanged.

For *N. caninum* the seroprevalence in sheep was of 0.8% (5/653; CI 95%: 0.3–1.8%) at animal level and of 2.8% (4/143; CI 95%: 0.8–7.0%) at the farm level, whilst the seroprevalence in goats was of 0.9% (7/748; CI 95%: 0.4–1.9%) and of 1.8% (3/164; CI 95%: 0.4–5.3%) at the animal and farm levels, respectively.

**Fig. 2:**
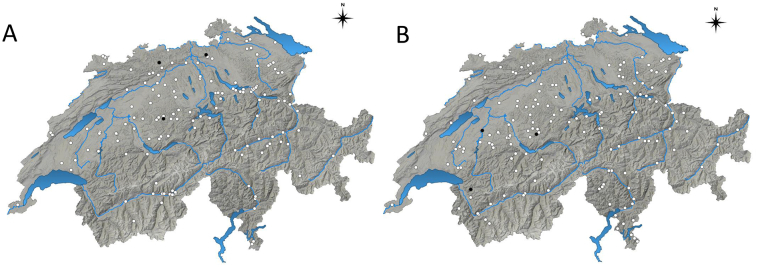
Geographical distribution of all tested farms breeding sheep (A) and goats (B) in Switzerland, indicating the farms in which seropositive animals for *Neospora caninum* were detected (black dots) or not detected (white dots).

Fig. 2 shows the distribution of all the tested sheep (A) and goat (B) farms in Switzerland, with black dots representing farms with a positive serological status for *N. caninum* and white dots for farms with a negative serological status.

### 3.2 Questionnaire and risk factor analysis

A total of 276 farms were invited to complete the web-based questionnaire (31 of the 307 farms did not provide an e-mail address and therefore no questionnaire could be sent), and only 43 (15.5%) of the farm owners completed the questionnaire, with 15 farms housing sheep, 23 farms housing goats and 5 farms housing both species. These 43 farms accounted for 220 out of all 1′401 (15.7%) animals tested in this study with 80/653 (12.3%) of the sheep and 140/748 (18.7%) of the goats, respectively. Considering only the responding farms, the total prevalence for *T. gondii* in small ruminants at the animal and farm level was 50.9% (112/220; CI 95%: 44.1–57.7%) and 81.4% (35/43: CI 95%: 66.6–91.6%), respectively.

Only one (2.3%) of the responding farms (housing both sheep and goats) reported breeding imported animals from Germany, all other farms had only animals that were born in Switzerland; 22 (51.1%) of the 43 farms (i.e. 66.7% (10/15) of the sheep farms, 39.1% (9/23) of the goat farms and 60% (3/5) of the farms breeding both ruminant species) reported that their animals were held on alpine pastures from late spring to early autumn each year. The presence of cats (either own or foreign cats) was reported from 74.4% (32/43) of the farms, and in all cases, the cats had access to the stables and/or pasture where small ruminants were held. Besides, a total of 65.1% (28/43) of the farms reported having housed kittens (younger than 6 months) in the last two years. The presence of dogs, either own or foreign, was reported from 69.8% (30/43) of all responding farms, and 76.7% (23/30) of these farms with dogs reported that the dogs had access to the small ruminants housing and/or pasture. Besides, 18.6% (8/43) of the farms reported having puppies and/or young dogs under the age of 6 months on their farm. Of all responding farms, 18.6% (8/43) reported having actual or past problems with rodents in their stables. A total of 90.7% (39/43) of the farms reported housing other species than sheep or goats (i.e., cattle, equids, South American camelids, cats, dogs, poultry, pigs) on their premises. The occurrence of abortions in the herd was reported from 39.5% (17/43) of the farms with only 4 (23.5%) of those 17 farms having submitted samples for diagnosis. In none of these 4 farms were *T. gondii* or *N. caninum* associated abortions recorded*.*

**Fig. 3:**
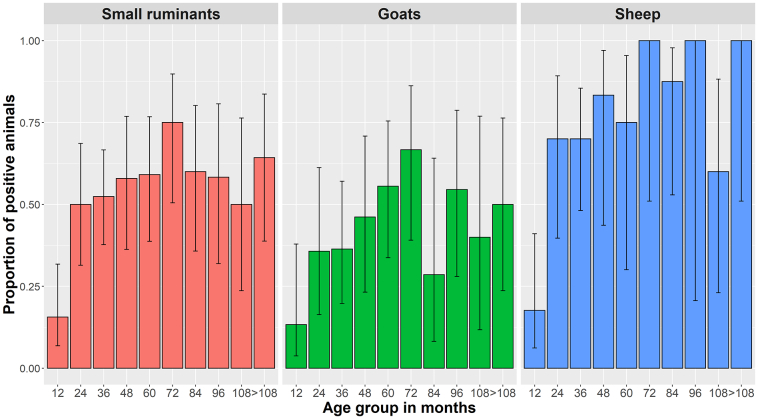
Proportion of sheep and goats seropositive to *Toxoplasma gondii* in different age categories.

A risk factor analysis for *T. gondii* seropositivity was performed by a bivariable generalized linear mixed modelling (LaPlace approximation) (Table 1); however, for *N. caninum* such analysis was not possible since none of the seropositive farms for *N. caninum* completed the questionnaire. The analysis included the factors “Age” (Age group young: ≤12 months-old; Age group adult: >12 months-old) as effect modifier and “Farm” as random effect variable in modelling individual *T. gondii*-seropositivity. Animals older than 12 months had a statistically significantly higher chance of being seropositive than younger animals in both small ruminant species. Besides, goats had a lower risk to become infected with *T. gondii* compared to sheep. The proportion of sheep and goats seropositive to *Toxoplasma gondii* in different age categories is displayed in Fig. 3. In addition, “grazing on alpine pastures” during the warm season was identified as a protective factor for *T. gondii* seropositivity in both sheep and goats. The factor “no equids on the farm” was also identified as a putative protective factor. For all further variables no statistical significance as risk or protective factor could be found.

**Table 1:** Fixed effects in generalized linear mixed models to determine potential risk factors for *Toxoplasma gondii*-seropositivity in Swiss sheep and goats. Data were analysed by bivariable generalized linear mixed modelling including “Age Group” (young [12 months and younger], adult [>12 months]) as effect modifier and “Farm” as random effect variable in modelling *T. gondii*-seropositivity. The Akaike information criterion (AIC) was used to characterize the relative model quality.

Model (AIC, model fit)	Variable	Odds ratio (95% CI)	*z-*value	*P*-value
1 (240.5)	(Intercept)	1.9231 (0.88312–4.188)	1.647	0.09956
	Age Group: adult (ref.)			
	Age Group: young	0.0371 (0.00663–0.208)	−3.745	0.00018 ***
2 (238.4)	(Intercept)	5.208 (1.49580–18.133)	2.593	0.00952 **
	Age Group: adult (ref.)			
	Age Group: young	0.034 (0.00626–0.184)	−3.920	8.85e-05 ***
	Species: sheep (ref.)			
	Species: goat	0.213 (0.04872–0.932)	−2.053	0.04003 *
3 (228.5)	(Intercept)	2.8423 (0.81210–9.948)	1.634	0.1022
	Age Group: adult (ref.)			
	Age Group: young	0.0243 (0.00379–0.156)	−3.918	8.91e-05 ***
	Species: sheep (ref.)			
	Species: goat	0.1760 (0.04330–0.715)	−2.428	0.0152 *
	Alpine pasturing: yes (ref.)			
	Alpine pasturing: no	4.5198 (1.18145–17.291)	2.204	0.0276 *
4 (232.4)	(Intercept)	12.3589 (2.74025–55.74)	3.272	0.00107 **
	Age Group: adult (ref.)			
	Age Group: young	0.0368 (0.00703–0.192)	−3.913	9.13e-05 ***
	Species: sheep (ref.)			
	Species: goat	0.1962 (0.04827–0.798)	−2.276	0.02286 *
	Equids on farm: yes (ref.)			
	Equids on farm: no	0.2293 (0.06030–0.872)	−2.161	0.03072 *
5 (222.4)	(Intercept)	5.4742 (1.28384–23.342)	2.298	0.02158 *
	Age Group: adult (ref.)			
	Age Group: young	0.0282 (0.00473–0.168)	−3.919	8.91e-05 ***
	Species: sheep (ref.)			
	Species: goat	0.1633 (0.04328–0.616)	−2.675	0.00748 **
	Alpine pasturing: yes (ref.)			
	Alpine pasturing: no	4.7253 (1.34318–16.623)	2.420	0.01553 *
	Equids on farm: yes (ref.)			
	Equids on farm: no	0.3083 (0.08780–1.082)	−1.837	0.06628

*Abbreviation*: ref., reference.

*P* ≤ 0.1, *P ≤ 0.05, **P ≤ 0.01, ***P < 0.001

### 3.3 Real-time PCR investigation of aborted ovine and caprine foetuses

*T. gondii* and *N. caninum* DNA were detected in 6.1% (5/82, 95%CI: 2.0–13.7%) and 2.4% (2/82; 95%CI: 0.3–8.5%) of the ovine aborted foetuses, and in 6.8% (5/73; 95%CI: 2.3–15.3%) and 1.4% (1/73; 95%CI: 0.0–7.4%) of the tested caprine foetuses, respectively. All positive foetuses derived from different farms, with exception of two ovine foetuses positive for *T. gondii* which derived from the same farm but were submitted two years apart. Farms with *T. gondii* or *N. caninum* positive ovine foetuses were located in the cantons of Bern, Argovia and Grisons, or Bern and Grisons, respectively. Farms with *T. gondii* or *N. caninum* positive caprine foetuses were located in the cantons of Fribourg, Ticino, St. Gallen and Valais, or Fribourg, respectively. None of these farms participated in the serosurvey.

## 4 Discussion

The aim of this study was to study the occurrence, distribution, and importance of *T. gondii* and *N. caninum* infections in small ruminants in Switzerland and to reveal risk factors that may favour infection with these parasites. Nevertheless, a risk factor analysis for seropositivity could be performed only for *T. gondii*, as only very few farms (i.e., 4 sheep and 3 goat farms) had seropositive animals for *N. caninum* and none of their owners answered to the web-based questionnaire.

*Toxoplasma gondii* infections were recorded in sheep and goat populations worldwide, with highly variable seroprevalences (Dubey, 2022). In this study, a high seroprevalence of *T. gondii* infection was revealed both in Swiss sheep (66.3%) and goats (50.5%), and farms with seropositive animals were distributed nationwide (Fig. 1 A and B). However, it seemed that in the southern, mountain region, goat farms had more frequently a seronegative status for *T. gondii* (Fig. 1 B). Our results are in line with recent observations in the neighbouring countries. In Italy, various studies were carried out during the last 10 years. In Lombardy, north Italy, an IFAT-based (cut-off: 1:64) serological study showed seroprevalences of 59.3% (298/502) and 41.7% (198/474) in sheep and goats of different ages, respectively, with prevalences at the farm level of 87.5% (21/24) and 96.6% (28/29) (Gazzonis et al., 2015). In Tuscany, central Italy, a seroprevalence of 34% (214/630) by IFAT (cut-off 1:64) was observed in adult dairy sheep from 32/33 (97%) flocks (Cenci-Goga et al., 2013). In the same region, a seroprevalence of 60.6% (77/127) was registered in adult dairy goats from 6/6 farms by MAT (cut-off 1:20) (Mancianti et al., 2013). In Southern Italy, a study in the Campania region performed with the same ELISA kit and conditions used in the present study revealed seroprevalences of 56.7% (221/390) in sheep and 47.3% (114/241) in goats of different ages, with farm prevalences of 93.1% (27/29) and 80.8% (21/26) respectively (Pepe et al., 2021). In France, a nationwide study based on meat juice analysis by MAT (cut-off: 1:6) showed seroprevalences of 15% in lambs and 81% in adult sheep (Halos et al., 2010). Age was regarded as a major risk factor for *T. gondii* seropositivity in small ruminants in numerous studies (Dubey, 2022; Stelzer et al., 2019). Accordingly, in a previous report from Switzerland, a higher *T. gondii* antibody prevalence was found in meat juice from slaughtered ewes compared with lambs (80.7% vs. 33%) (Berger-Schoch et al., 2011), and also in the present study “older age” was recognised as a risk factor for seropositivity in both studied animal species, and a higher seroprevalence was observed in older sheep and goats. This fact highlights the importance of postnatal transmission and suggests that the environmental contamination with *T. gondii* oocysts in sheep and goat breeding areas in Switzerland is high. Besides, we recorded a significant higher *T. gondii* seroprevalence in sheep than in goats (i.e., 66.3% vs. 50.5%) and revealed that goats have a lower risk of being seropositive than sheep. Higher *T. gondii* seroprevalences in sheep than in goats were also observed in other studies, e.g., in Italy (IFAT, cut-off: 1:64): 59.3% in sheep vs. 41.7% in goats (Gazzonis et al., 2015); Spain (ELISA): 49.3% in sheep vs. 25.1% in goats (García-Bocanegra et al., 2013), and (MAT, cut off 1:25): 46.5% in sheep vs. 38.3% in goats; Greece (ELISA): 48.6% in sheep vs. 30.7% in goats (Tzanidakis et al., 2012); Portugal (MAT, cut-off 1:20): 33.6% in sheep vs. 18.5% in goats (Lopes et al., 2013) and Poland (ELISA): 47% in sheep vs. 21% in goats (Moskwa et al., 2018). The observed differences in the seroprevalence between sheep and goats could be associated to the different grazing habits of both small ruminant species. Since sheep are grazers that eat short plants near to the ground, they are more likely to ingest oocysts contaminating grass and soil than goats that are mainly browsers, and prefer leaves, shrubs, and vines, which would make up to 60% of their daily diet if the opportunity is given. A similar situation was observed in regard to *N. caninum* infection, in which a higher seroprevalence was observed in sheep than in goats in Italy (Gazzonis et al., 2016), and to anthrax in ruminants, where it was assumed that cattle and sheep tend to be more commonly affected than goats due to their different grazing habits (Underwood et al., 2015). Grazing on alpine pastures during the warm season was shown to be a significant protective factor for both sheep and goats in this study. This might be attributed to a lower density of cats in mountain regions, far from the farm dwellings, accounting for a lower environmental contamination with *T. gondii* oocysts. Besides, other factors such as altitude, extreme low temperatures and exposure to ultraviolet rays may negatively influence oocysts survival. Accordingly, also a very low prevalence of antibodies against *T. gondii* was registered in Alpine ibex (*Capra ibex ibex*) from 14 colonies throughout the Swiss Alps (i.e., 0.9%; 95%CI 0.3–2.1) (Marreros et al., 2011). “Absence of equids in the farm” was also suggested as a putative protective factor for *T. gondii* seropositivity, but no clear explanation for this possible association could be found.

Some studies observed cross reactions of *N. caninum* antibodies against *T. gondii* TgSAG1 antigen (Huertas-López et al., 2021; Sánchez-Sánchez et al., 2021a). However, this issue seems to be irrelevant in this study, in which only a very low proportion of seropositive animals to *N. caninum* were recorded. Moreover, we observed that although 4 out of 5 sheep seropositive to *N. caninum* were also positive for *T. gondii* in ELISA, only 1 out of 7 goats seropositive for *N. caninum* were seropositive for *T. gondii*, suggesting that putative cross reactions were not relevant.

Toxoplasmosis is one of the most frequent food-borne zoonoses worldwide and can lead to a life-threatening disease in immunosuppressed patients and congenitally infected new-borns (Dubey, 2022). Meat from infected sheep, goats, pigs, as well as game are regarded as an important source of infection for humans when consumed raw or undercooked (Cook et al., 2000; Kapperud et al., 1996; Edelhofer and Prossinger, 2010). Besides, oocysts shed by cats are of utmost importance as source of infection for herbivores (Dubey, 2022). The consumption of sheep and goat meat in Switzerland stayed relatively stable during the past ten years and is well below the consumption of pork, poultry and beef (Leuenberger, 2019). But taking into account the high seroprevalence of *T. gondii* in sheep and goats found in the present study, and the fact that isolation of viable parasites from tissues of seropositive small ruminants is often successful (Juránková et al., 2015), the risk for consumers, and especially for pregnant women and immunosuppressed patients should not be underestimated and treatment of the meat for parasite inactivation (e.g., freezing at < −12 °C for at least two days before consumption, cooking with core temperature ≥ 66 °C) (Dubey, 2022) is recommended. Besides, consumption of raw milk or dairy products from infected sheep and goats, such as fresh cheese made by cold-enzyme treatment, represents a risk of infection for humans and animals, as excretion of *T. gondii* in the milk of naturally and experimentally infected small ruminants was reported and isolation of *T. gondii* from goat milk by bioassays in mice and cats was successful (Mancianti et al., 2013; Dubey, 2022).

In addition, *T. gondii* is well recognised as a major infectious cause of abortion and neonatal mortality in small ruminants worldwide (Dubey, 2022; Buxton et al., 2007). In this study, *T. gondii* DNA was found in 6.1% (5/82, 95%CI: 2.0–13.7%) and 6.8% (5/73; 95%CI: 2.3–15.3%) of the tested ovine and caprine aborted foetuses, respectively, documenting the occurrence of congenital transmission and its potential association with abortion in sheep and goats. However, histological examination to search for associated lesions and exclusion of other abortifacients would be needed to establish the definitive cause of abortion. The same applies for *N. caninum*.

In a previous study investigating the main abortifacient agents in small ruminants in Switzerland, *T. gondii* was the second most frequently detected agent (after *Chlamydophila abortus*), being revealed by immunohistochemical staining (IHC) in 19% (16/86) and 15% (22/144) of the tested aborted ovine and caprine foetuses, respectively (Chanton-Greutmann et al., 2002). In the mentioned study, *N. caninum* was not detected by IHC in any of the foetuses.

*Neospora caninum* is well recognised as a major abortifacient in cattle (Dubey et al., 2017) and although it seems to have a lower clinical and economic importance in small ruminants, there is increasing evidence of its involvement in cases of abortion and reproductive failure both in sheep and goats worldwide (Moreno et al., 2012; Della Rosa et al., 2021; Sánchez-Sánchez et al., 2021a, Sánchez-Sánchez et al., 2021b; Unzaga et al., 2014; Dubey et al., 2017; Hässig et al., 2003).

In the present study, the observed seroprevalences for *N. caninum* were 0.8% in sheep and 0.9% in goats, and seropositive animals were detected in only few farms (i.e., in 7 out of 307 Swiss farms breeding small ruminants). These seroprevalences were much lower than those of antibodies against *T. gondii* in both species (i.e., 66.3% and 50.5% in sheep and goats, respectively). High seroprevalences for *T. gondii* and lower antibody prevalences for *N. caninum* were also found in other European countries when testing small ruminants simultaneously for both parasites: i.e., in Greece (Diakou et al., 2013), Romania (Iovu et al., 2012), Slovakia (Spilovská et al., 2009; Čobádiová et al., 2013) and Spain (Díaz et al., 2016; Ruiz-Fons et al., 2014), suggesting a general lower exposition to *N. caninum*. In Switzerland there are more than three times more domestic cats than dogs registered (1,634,000 vs. 506,000 in 2018) (Schultz, 2019) (https://de.statista.com/themen/3748/haustiere-in-der-schweiz/), which could account for a potential higher environmental contamination with *T. gondii* than with *N. caninum* oocysts. Furthermore, the reported frequencies of cats shedding *T. gondii* oocysts in Switzerland were between 0.4% (Frey et al., 2012) and 0.6% (Schreiber et al., 2021), which is probably >10 times higher than the frequency of dogs shedding *N. caninum* oocysts (Basso et al., 2020). So far, only few studies on *N. caninum* seroprevalence in small ruminants have been reported in Europe (summarized in Table 2, Table 3), and a direct comparison is difficult due to the different diagnostic tests and cut-offs used. However, it seems that the prevalences of *N. caninum* antibodies in healthy sheep and goats in Switzerland (being lower than 1%) are amongst the lowest in Europe (Table 1, Table 2). In a previous study from Switzerland, a higher *N. caninum* seroprevalence of 10.3% (12/117) was reported in sheep; nevertheless, that prevalence was assessed in a flock with a history of abortion, in which *N. caninum* was effectively detected in aborted ovine foetuses (Hässig et al., 2003). The lower seroprevalence observed in this study can be attributed to the fact that all samples derived from clinically healthy sheep, chosen from the flock randomly.

**Table 2:** Prevalence of antibodies against *N. caninum* in sheep in Europe.

Country	Region	Test (source)	n positive animals/ n examined animals	% positive	n farms with positive animals/ n sampled farms	% positive	Observations	Reference
Czech Republic	Central Bohemia Ústí nad Labem	ELISA (cELISA Neospora caninum Antibody Test Kit, VMRD, USA)	63/547	12.0	9/9	100	Healthy adult sheep.	(Bártová et al., 2009)
Greece	Various regions	ELISA (in house)	77/458	16.8	28/50	56.0	Healthy dairy ewes (age 2–4 years), semi-extensive grazing, with grain and forages supplementation	(Diakou et al., 2013)
Italy	Lombardy: Bergamo, Milan, and Varese provinces	ELISA (in house) + immunoblot as confirmatory test	83/428	19.3	ns	89.4	Randomly sampled general sheep population, different rearing systems (semi-extensive, transhumant)	(Gazzonis et al., 2016)
	Orobie Alps, Bergamo province	ELISA (CHEKIT Neospora, Bommeli Diagnostics, Switzerland)	22/1010	2.2	ns	ns	Randomly sampled general sheep population sharing habitat with wild ungulates during warm season	(Gaffuri et al., 2006)
Poland	Mazurian lake district	ELISA (cELISA Neospora caninum Antibody Test Kit, VMRD, USA) + immunoblot as confirmatory test	8/64	13.0	1/1	100	Healthy Merino ewes (age 1–2 years) from a mixed flock with goats and fallow deer, semi-extensive management	(Moskwa et al., 2018)
Slovakia	Kosice, Presov	ELISA (Neospora caninum indirect ELISA ID.vet, France) + verification of positive and doubtful sera by cELISA (cELISA Neospora caninum Antibody Test Kit, VMRD, USA).	14/382	3.7	ns/100	ns	Dairy ewes, which had aborted	(Spilovská et al., 2009)
Spain	Castilla y León: Zamora province	ELISA (NcSALUVET ELISA, Spain)	304/986	30.8	1/1	100	Dairy Assaf breed ewes with history of abortion in the flock, semi-intensive management, balanced ration (concentrate and forage) feeding. *N. caninum* seropositive dams were more likely to abort and to have repeated abortions than seronegative ones. *N. caninum* DNA was isolated from aborted foetuses.	(Sánchez-Sánchez et al., 2021a, Sánchez-Sánchez et al., 2021b)
	Extremadura: Badajoz, and Andalusia: Córdoba and Jaén	ELISA (ID Screen Neospora caninum Indirect Multispecies, ID.vet, France)	4/209	1.9	3/12	25.0	Culled Merino and crossbred ewes with no particular disease, extensive breeding systems	(Astorga et al., 2014)
	Castilla-La Mancha: Ciudad Real province	ELISA (cELISA Neospora caninum Antibody Test Kit, VMRD, USA)	7/180	3.9	ns/17	ns	Manchega sheep flocks, male and female sheep	(Ruiz-Fons et al., 2014)
	Galicia	ELISA (ID Screen Neospora caninum Indirect Multi-species, ID.vet, France)	132/2400	5.5	32/44	72.7	Healthy adult sheep (> 6 months) from lamb-producing flocks	(Díaz et al., 2014)
	Galicia: Lugo Province	ELISA (cELISA Neospora caninum Antibody Test Kit, VMRD, USA)	18/177	10.1	ns	ns	Meat crossbred ewes, semi-extensive breeding system, grazing with hay or silage supplement	(Panadero et al., 2010)
Switzerland	Country-wide	ELISA (ID Screen® Neospora caninum Indirect, ID.vet, France) + immunoblot (in house) of inconclusive sera	5/653	0.8	4/143	2.8	Randomly sampled general sheep population, healthy animals, different sex and ages	This study
	Zurich	IFAT (in house, cut-off 1:160)	12/117	10.3	1/1	100	Ewes from a flock with abortion problems. *N. caninum* DNA detected in aborted foetuses	(Hässig et al., 2003)
United Kingdom	England and Wales	ELISA (Mastazyme, Mast Diagnostics) + verification of positive sera by IFAT (in house, cut-off 1:50)	3/660	0.45	ns	ns	Ewes, which had recently aborted, sent by veterinary practitioners	(Helmick et al., 2002)

ELISA: Enzyme-linked immunosorbent assay; cELISA: competitive ELISA; IFAT: indirect fluorescent antibody test; ns: not specified.

**Table 3:** Prevalence of antibodies against *N. caninum* in goats in Europe.

Country	Region	Test (commercial provider)	n positive animals/ n animals examined	%	n farms with positive animals/ n sampled farms	%	Observations	Reference
Czech Republic	Karlovy Vary, Usti Labem, Central Bohemia, Liberec, Prague, Hradec Kralove, Plzen, and Pardubice	ELISA (cELISA Neospora caninum Antibody Test Kit, VMRD, USA) + IFAT (VMRD, USA, cut-off 1:40) as confirmatory test	15/251	6.0	ns/15		healthy adult goats (age ≥ 12 months)	(Bártová and Sedlák, 2012)
Greece	Various regions	ELISA (in house)	26/375	6.9	28/50	56	Healthy adult dairy female goats, semi-extensive grazing, with grain and forages supplementation	(Diakou et al., 2013)
Italy	Lombardy: Bergamo, Milan, and Varese provinces	ELISA (in house) + immunoblot as confirmatory test	24/414	5.7	ns	32.1	Randomly sampled goat population, different rearing systems (intensive, semi-extensive, transhumant)	(Gazzonis et al., 2016)
Poland	Mazurian lake district	ELISA (cELISA Neospora caninum Antibody Test Kit, VMRD, USA) + immunoblot as confirmatory test	8/39	21.0	1/1	100	Healthy adult Polish Fawn Improved female goats (age 1–2 years) from a mixed flock with sheep and fallow deer, semi-extensive management	(Moskwa et al., 2018)
	Country-wide	ELISA (CHEKIT Neospora caninum Antibody ELISA, IDEXX) + IFAT (VMRD, USA, cut-off 1:160) as confirmatory test	5/1060	9.0	4/49	0.8	Adult dairy female goats (≥12 months)	(Czopowicz et al., 2011)
Romania	Crişana, Maramures¸ Transylvania and Muntenia	ELISA (Chekit Neospora caninum Antibody ELISA; IDEXX-Bommeli, Switzerland)	12/512	2.3	ns	ns	dairy female goats (kids and adult goats), backyard and semi-extensive management, grazing and supplemented with fodder and concentrates	(Iovu et al., 2012)
Slovakia	Eastern region	ELISA (cELISA Neospora caninum Antibody Test Kit, VMRD, USA)	18/116 (a) 6/41 (b)	15.5 (a) 14.6 (b)	1/1	100	(a) adult dairy White Shothaired female and male goats (age 1–4 years) from farm with history of frequent abortions, semi-extensive management. (b) kids (< 6 months). *N. caninum* DNA was detected in blood from 14/18 *Neospora*-seropositive animals	(Čobádiová et al., 2013)
Spain	Galicia	ELISA (cELISA Neospora caninum Antibody Test Kit, VMRD, USA)	45/638	6.0	19/50	38.0	Male and female Cabra Galega and crossbred goats, different ages, extensive and semi-extensive husbandry system	(Díaz et al., 2016)
Switzerland	Country-wide	ELISA (ID Screen® Neospora caninum Indirect (ID.vet, France) + immunoblot (in house) of inconclusive sera	7/748	0.9	3/164	1.8	Randomly sampled general goat population, healthy animals, different sex and ages	This study

ELISA: Enzyme-linked immunosorbent assay; cELISA: competitive ELISA; IFAT: indirect fluorescent antibody test; ns: not specified.

In addition, *N. caninum* DNA was detected in 2.4% (2/82; 95%CI: 0.3–8.5%) and 1.4% (1/73; 95%CI: 0.0–7.4%) of the tested sheep and goat foetuses, respectively, showing occurrence of vertical transmission of this parasite and suggesting its involvement as cause of abortion. Although the frequency of *N. caninum*-DNA detection in small ruminants was around ten times lower than that previously reported for Swiss cattle, in which *N. caninum* DNA was amplified in 29% (24/83) (Gottstein et al., 1998) and 21% (50/242) (Sager et al., 2001) of aborted bovine foetuses, this parasite was shown to cause important reproductive losses in some sheep flocks in Switzerland (Hässig et al., 2003) as well as in other countries (Della Rosa et al., 2021; Sánchez-Sánchez et al., 2021a, Sánchez-Sánchez et al., 2021b; González-Warleta et al., 2018; Moreno et al., 2012); therefore, *N. caninum* should be also considered in the differential diagnosis of reproductive failure in small ruminants.

## 5 Conclusion

This nationwide cross-sectional study identified a high prevalence of antibodies against *T. gondii* and a low prevalence of antibodies to *N. caninum* in the Swiss sheep and goat population and shed some light on the epidemiological situation of these parasites. Our results suggest that the consumption of meat or raw milk from *T. gondii* infected sheep and goats might represent a risk for public health. Furthermore, infection with both parasites was detected in aborted ovine and caprine foetuses by molecular methods, suggesting their potential involvement in cases of abortion or reproductive failure in small ruminants. As both toxoplasmosis and neosporosis are reportable animal diseases in Switzerland, our results provide valuable information to be considered in current control programs.

## Funding

This research did not receive any specific grant from funding agencies in the public, commercial, or not-for-profit sectors.

The following are the supplementary data related to this article.

Suppl. Table 1 Collected data to identify putative risk factors for *T. gondii* and *N. caninum* seropositivity in Swiss sheep and goats. Suppl. Table 1

## Declaration of Competing Interest

The authors declare that they have no competing interests or personal relationships that could have appeared to influence the work reported in this paper.
