# Supplementary material for: Toxoplasma gondii and Neospora caninum infections in sheep and goats in Switzerland: Seroprevalence and occurrence in aborted foetuses
Source: Food Waterborne Parasitol. 2022 Aug 17;28:e00176. doi: 10.1016/j.fawpar.2022.e00176 (PMC9418186; doi:10.1016/j.fawpar.2022.e00176)
Supplement: Suppl. Table 1 — Collected data to identify putative risk factors for T. gondii and N. caninum seropositivity in Swiss sheep and goats. [file mmc1.docx]

| **Suppl. Table 1:**  **Collected data to identify putative risk factors for *T. gondii* and *N. caninum* seropositivity in Swiss sheep and goats** |
| --- |
| - Farm: location, identification number, contact data - Small ruminant species, number of animals in the herd, breed, origin, purpose of use (e.g., milk, meat, hobby, wool, other) |
|  |
| - Inclusion of a grazing period in alpine pastures during the warm season (“summering”) - Presence of cats/dogs in the stables and pasture - Presence of kittens and puppies younger than six months-old within the last two years |
|  |
| - Problems with rodents (mice or rats) on the farm |
| - Presence of other animal species on the farm |
| - Type of water supply (e.g., creek, water from own well, tap water, groundwater) |
|  |
|  |
| - Type of feed (e.g., pasture, hay, silage, commercial concentrate, migrating flock) |
| - Type of feed storage (e.g., hayrick, hay balls, foliated hay balls, silo balls, horizontal silo, vertical silo) |
| - Occurrence of abortions in the herd during last two years - Sending of abortion material (placenta, foetus) for investigation - Identification of any abortion cause in the farm - Disposal of abortions |
|  |
